# Downscaling patterns of complementarity to a finer resolution and its implications for conservation prioritization

**DOI:** 10.1002/ece3.2190

**Published:** 2016-05-18

**Authors:** Fábio Suzart de Albuquerque, Paul Beier

**Affiliations:** ^1^ Science and Mathematics FacultyCollege of Letters and SciencesArizona State UniversityMesaArizona85212; ^2^ School of ForestryNorthern Arizona UniversityFlagstaffArizona86001

**Keywords:** Biological conservation, prioritization, protected areas, rarity, richness, surrogate, zonation

## Abstract

Given species inventories of all sites in a planning area, integer programming or heuristic algorithms can prioritize sites in terms of the site's complementary value, that is, the ability of the site to complement (add unrepresented species to) other sites prioritized for conservation. The utility of these procedures is limited because distributions of species are typically available only as coarse atlases or range maps, whereas conservation planners need to prioritize relatively small sites. If such coarse‐resolution information can be used to identify small sites that efficiently represent species (i.e., downscaled), then such data can be useful for conservation planning. We develop and test a new type of surrogate for biodiversity, which we call downscaled complementarity. In this approach, complementarity values from large cells are downscaled to small cells, using statistical methods or simple map overlays. We illustrate our approach for birds in Spain by building models at coarse scale (50 × 50 km atlas of European birds, and global range maps of birds interpreted at the same 50 × 50 km grid size), using this model to predict complementary value for 10 × 10 km cells in Spain, and testing how well‐prioritized cells represented bird distributions in an independent bird atlas of those 10 × 10 km cells. Downscaled complementarity was about 63–77% as effective as having full knowledge of the 10‐km atlas data in its ability to improve on random selection of sites. Downscaled complementarity has relatively low data acquisition cost and meets representation goals well compared with other surrogates currently in use. Our study justifies additional tests to determine whether downscaled complementarity is an effective surrogate for other regions and taxa, and at spatial resolution finer than 10 × 10 km cells. Until such tests have been completed, we caution against assuming that any surrogate can reliably prioritize sites for species representation.

## Introduction

Prioritization algorithms identify sets of sites that efficiently represent species (Stein et al. 2000; Ferrier and Wintle 2009; Moilanen et al. 2009, 2014). A solution (set of sites) is optimum if it represents the largest possible number of species in a specified number of sites (or alternatively requires the fewest number of sites to represent all species). Several procedures produce optimum or near optimum solutions; these include the simulated annealing algorithm in the software program Marxan (Ball et al. 2009), the reverse stepwise heuristic algorithm in program Zonation (Moilanen et al. 2009), and rarity‐weighted richness, RWR (Williams et al. 1996; Csuti et al. 1997; Albuquerque and Beier 2015a). Each of these procedures ranks each site in terms of its contribution to the goal of species representation. We refer to this rank as the complementarity value of the site because it indicates how much each site complements (adds unrepresented species to) the other sites in the solution set.

Although fine‐scale biodiversity data are becoming more widely available, wall‐to‐wall species inventories (i.e., inventories of all sites available for selection) do not exist for any planning area and such data would be expensive to acquire (Hurlbert and Jetz 2007; Venter et al. 2014). Therefore, the utility of conservation algorithms is limited because distributions of species are typically available only as coarse atlases or range maps, whereas conservation planners need to prioritize relatively small sites. For example, the Atlas of European Breeding Birds lists bird species present in each 50 × 50 km cell in Western and Central Europe (Hagemeijer and Blair 1997) and BirdLife International (2012) provides range maps of all bird species. If such coarse‐scale information can be downscaled (i.e., used to identify high‐priority sites at local scales), then such data can be useful for conservation planning.

One intuitively appealing way to downscale coarse data is to build species distribution models (SDMs) for each species as a function of environmental conditions in coarse atlas cells and use the resulting model to predict occupancy at finer cell size (when sites are squares on a grid, the terms *cell* and *site* are synonyms). Then, one can apply a prioritization algorithm to select a complementary set of sites. Araújo et al. (2005) took this approach. In their study, SDMs built using 50 × 50 km atlas data were only moderately successful in predicting species distributions in 10 × 10 cells. Furthermore, the 10 × 10 km cells with the highest expected complementarity (as predicted from downscaled data) overlapped only 23% (birds) to 47% (plants) of the cells with highest true complementarity. Newer downscaling procedures (e.g., Azaele et al. 2012; Keil et al. 2013; Barwell et al. 2014) yield more accurate predictions of species occupancy at fine scales, but have not been evaluated for their ability to identify sites with high complementarity values. Moreover, SDMs cannot be developed for species that occur in only a few of the coarse cells. For example, Araújo et al. (2005) did not build models for the 28% of species that occurred in the fewest cells, and Keil et al. (2013) similarly could not build models for about 14% of species. Thus, downscaling SDMs can overlook the range‐restricted species that are often the focus of conservation efforts.

We tried a different approach to downscaling, namely downscaling complementarity value from large cells to small cells, using statistical methods or simple map overlays. This approach can be applied to any measure of complementarity value, such as selection frequency in Marxan, importance score in Zonation, or RWR rank. We chose to use RWR because RWR can be computed much faster and easier than other measures of complementarity, and its complementarity scores are highly correlated (in rank order) with complementarity scores produced by more complex procedures (Csuti et al. 1997; Albuquerque and Beier 2015a).

In this article, we demonstrate that downscaled complementarity efficiently identifies fine‐grain cells with high complementary. We used two methods to downscale RWR, namely statistical downscaling (of coarse atlas data and global range maps) and direct downscaling (of global range maps only). In statistical downscaling, we measured complementarity (RWR) in large (50 × 50 km) cells, modeled complementarity as a function of environmental conditions in those coarse cells, and used the resulting model to predict complementarity for smaller cells (10 × 10 km). In direct downscaling, we counted the number of species ranges that overlapped each 10‐km grid cell, calculated the number of 10‐km cells overlapped by each species range, and calculated RWR for each cell. We illustrate our approach for birds in Spain by building models using coarse data (a 50 × 50 km atlas of European birds, or global range maps of birds interpreted at the same 50 × 50 km grain size), and using these models to predict complementary value for 10 × 10 km cells in Spain. Then, we used an independent atlas of Spanish birds in those 10 × 10 km cells to assess how efficiently downscaled complementarity represented the birds of Spain, as measured by the Species Accumulation Index (SAI – Rodrigues and Brooks 2007). SAI assesses the surrogacy value by comparing the number of species represented in sites selected to represent the surrogate, to the largest number of species that can be represented in the same number of sites and to the number of species represented in the same number of randomly selected sites.

## Materials and Methods

### Data acquisition and preparation

**Figure 1:**
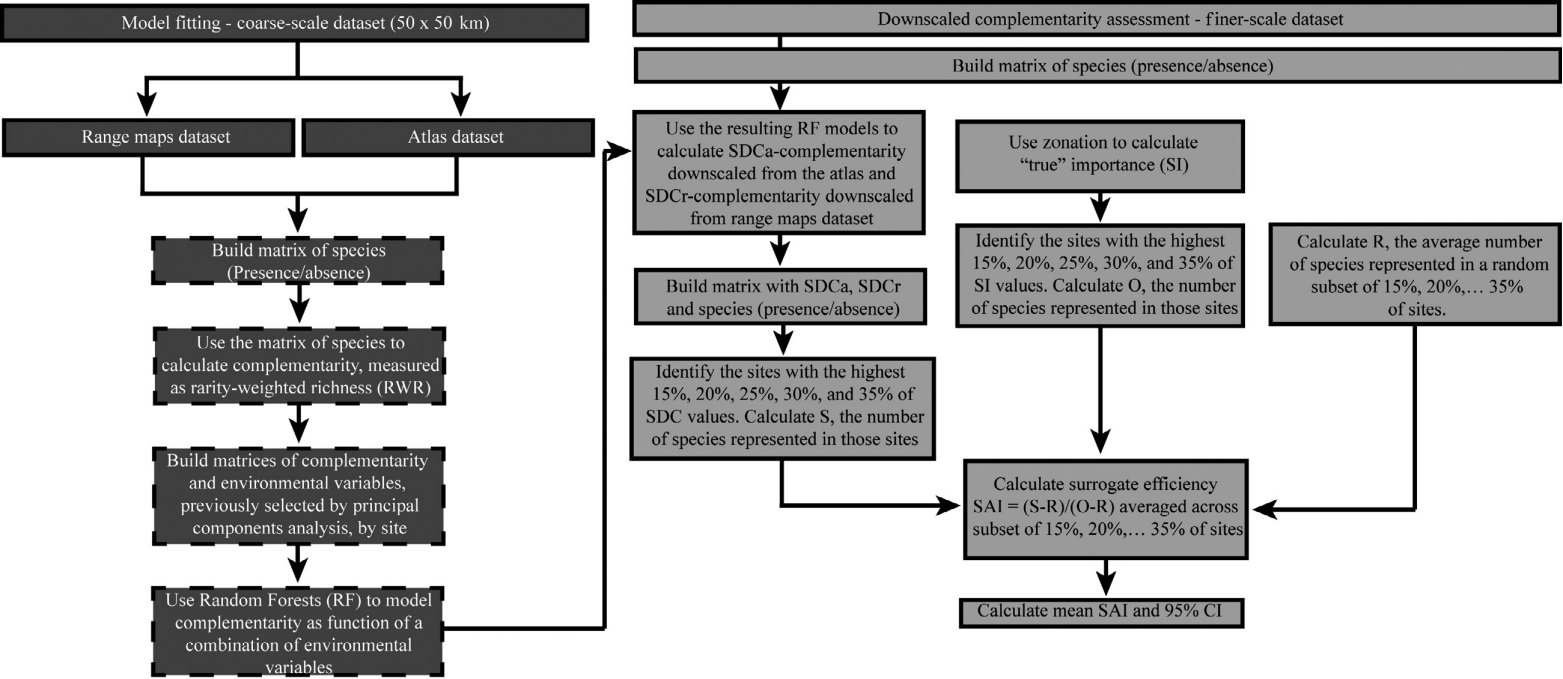
Illustration of steps taken to (dark boxes) model complementarity value as a function of environmental variables using coarse‐scale dataset (50 × 50 km), and (light boxes) use of the resulting coarse‐scale model to calculate complementarity values for a fine‐scale dataset (10 × 10 km) and test how well sites prioritized in order of downscaled complementarity incidentally represent species. Boxes with dashed borders indicate steps that are repeated for range maps and atlas dataset.

We downscaled 2 coarse‐scale datasets, namely the Atlas of European Breeding Birds (Hagemeijer and Blair 1997), which summarizes presence of bird species in a grid of ~50 × 50 km cells, including 267 species in 241 cells in Spain, and global bird range maps from BirdLife International (2012), which include 322 bird species range maps that overlap Spain. We processed the range maps to generate presence values for each 50‐km grid cell at least partially overlapped by a species range map. We evaluated how well complementarity downscaled from these coarse sources reflected complementarity in the independent fine‐grain (10 × 10 km) Spanish Bird Atlas (Martí and Del Moral 2003; INB 2007), which includes records of 263 native bird species in 5303 cells in mainland Spain (Fig. 1).

We selected 37 potential predictor variables associated with species richness patterns. We obtained temperature and precipitation variables from Hijmans et al. (2005), NDVI (normalized difference vegetation index) from Tucker et al. (2004), elevation and slope from USGS (n.d.), potential evapotranspiration and precipitation variables from Zomer et al. (2008), sunshine and topographic diversity (Benito et al. 2013) from USGS (n.d.). We calculated the mean or range of each environmental variable across each 50‐km and 10‐km grid cell.

### Identifying environmental gradients

We used varimax‐rotated factor analysis (VrFA), across the 241 50‐km cells, to identify major environmental gradients and identify a set of environmental variables with low multicollinearity. We used the Kaiser criterion (Kaiser 1960) to select PCA factors with an eigenvalues >1. We then identified the environmental variable that was most highly correlated with each significant factor. Because PCA factors are by definition orthogonal to each other, these environmental variables tend to have low multicollinearity.

### Modeling complementarity as a function of environmental variables

Williams et al. (1996) proposed that the rarity value of a species can be characterized by the inverse of the number of cells in which it occurs. Thus, if a species is found in only one cell, the species would have the maximum rarity score of 1/1 = 1, and a species that occurs in 20 cells would have a rarity score of 1/20 = 0.05. Williams et al. also proposed that the rarity scores of all species in a cell can be summed to yield a single rarity‐weighted richness value for the cell, RWR = $\sum_{i=1}^{n}\frac{1}{\left(c_{i}\right)},$ where *c*_*i*_ = the number of cells in which species *i* occurs and the values are summed for the *n* species occurring in that cell. RWR has been demonstrated to reliably indicate complementarity value of cells in all datasets tested (Csuti et al. 1997; Albuquerque and Beier 2015a).

For each of the two coarse‐grain datasets, we developed a random forests model, with RWR of the 50‐km cell as dependent variable, and the selected environmental variables measured in those 50‐km cells as independent variables. Random forests (Breiman 2001) is a machine learning method based on an ensemble of regression trees. To build our random forests models, we first randomly drew 500 bootstrap samples, each consisting of about 66% of the data. We used these samples to develop 500 regression trees, in each case choosing the best split among a given number of predictors. The remaining data (about 33%) were used to estimate error rate based on the training data (out‐of‐bag [OOB] error). The OOB error was then used to estimate the relative importance of the predictors by observing how much the OOB error changes when the values for a particular predictor were permuted in the training set while all other predictor values were left unchanged. Specifically, the predictor error on the OOB data was calculated for each tree and for each predictor variable. The importance score for each variable is the mean difference between in OOB error before and after permutation. For each random forests model, we evaluated 500 trees, which is substantially beyond the number of trees (about 200) at which mean squared error declined below 0.05.

### Statistically downscaled complementarity, SDC_a_, and SDC_r_

We applied the fitted random forest models to the same environmental variables measured in each 10‐km grid cell to downscale RWR (DRWR) for each 10‐km cell. This procedure generated two sets of DRWR values: (1) SDC_a_: calculated using the fitted random forest models from coarse‐grain atlas data; and (2) SDC_r_: calculated using the fitted random forest model from the range maps.

### Directly downscaled complementarity, DDC_r_

We overlaid the global range maps over the 10 × 10 km grid to the fine‐grain atlas data, counted the number of species ranges that overlapped each 10‐km grid cell, calculated the number of 10‐km cells overlapped by each species range, calculated RWR for each cell, and used this RWR value as DDC_r_.

### Evaluating the performance of downscaled complementarity as a surrogate for species representation

We used the Species Accumulation Index, SAI (Ferrier and Watson 1997; Rodrigues and Brooks 2007), to evaluate the efficiency of SDC_a_, SDC_r_, and DDC_r_ as surrogates to identify sites that represent Spanish birds in 10‐km grid cells. SAI compares the number of species represented in the set of sites selected using DRWR, to an optimum value *O* (the largest number of species that can be represented in the same number of sites) and to *R*, the mean number of species represented in the same number of randomly selected sites.

We used two procedures to estimate *O* for the 10‐km bird atlas data, namely the basic core area formulation of the reserve selection software Zonation (Moilanen et al. 2014) and RWR. Both procedures produced identical values of *O*; here, we report *O* from Zonation results. Zonation starts with all cells hypothetically “reserved” and iteratively removes cells that are least needed to maintain core areas of each species. The algorithm minimizes biological loss by minimizing proportional loss of geographic range (number of sites) for the worst‐off species (those species with the smallest remaining range in the current tentative solution). This produces a hierarchy of sites in which the most important 5% is a subset of the most important 10%, and so on. Cells receive a score between 0 and 1; values close to one indicate cells removed in the last state of the process, whereas values close to 0 indicate cells removed early.

To calculate *S*, we accumulated cells starting with the cell with the highest value of the surrogate (SDC_a_, SDC_r_, or DDC_r_), sequentially adding the cell with the next highest downscaled complementarity value. As cells were accumulated, the number of species represented in at least one cell was calculated. To calculate *R*, we accumulated cells in random order and at each step we calculated the number of species represented at least once in the randomly selected cells. We repeated the random selection procedure 1,000 times and used the mean value as *R*.

Formally, SAI = (*S–R*)/(*O–R*). SAI is scaled −∞ to 1; negative SAI indicates a worse than random result, 0 indicates random performance, and positive SAI is a measure of surrogate efficiency. SAI is sometimes calculated using the entire area under the *S*,* O*, and *R* curves. We used an alternative procedure, calculating SAI at 15%, 20%, 25%, 30%, 35% of the landscape hypothetically reserved. We chose this procedure to reflect performance of each surrogate at various plausible levels of a protected area network. We used the mean across these five levels of protection as the point estimate for SAI.

To examine the extent to which prioritizations differed among SDC_a_, SDC_r_, DDC_r_, and “true” priority based on the most complementary 10‐km cells, we calculated the Pearson correlation coefficients among SDC_a_, SDC_r_, and DDC_r_ and complementarity values of 10‐km cells. Significance values were corrected for spatial autocorrelation using a modified *t*‐test proposed by Dutilleul (1993).

All analyses were performed within GRASS (GRASS GIS 6.4, GRASS Development Team, 2012) and R (R Development Core Team, 2009).

## Results

The varimax‐rotated factor analysis (VrFA) identified six significant environmental gradients across the 241 coarse (50 km) cells; the most heavily loaded factors on the six axes were precipitation seasonality, minimum NDVI, precipitation of wettest quarter, hours of sunshine maximum, isothermality, and NDVI interquartile range (Table S1). The random forests models relating SDC_a_ and SDC_r_ to these six variables explained 16% and 50% of the variation in the dependent variable, respectively.

All three forms of downscaled complementarity were effective surrogates for identifying 10‐km grid cells that represented native Spanish birds. The two statistically downscaled surrogates were somewhat more effective than the directly downscaled surrogate. Complementarity statistically downscaled from global range maps, SDC_r_, had a mean efficiency of 77% (Table 1), meaning that it was 77% as effective as having the full knowledge of species occurrence in all sites in its ability to improve on random selection of sites. SDC_a_ was almost as effective, with an efficiency of 73%. Directly downscaled complementarity had a mean efficiency of 63%. SDC_a_, SDC_r_, and DDC_r_ were only moderately correlated with each other (Table 2).

**Table 1:** Species Accumulation Index (SAI) for three types of downscaled complementarity used to prioritize sites to represent all native bird species in Spain. SAI values indicate how efficiently downscaled complementarity represented species compared to the same number of randomly selected sites and the largest number of species that could be represented in the same number of sites

% of sites	Downscaled complementarity		
	SDC_a_	SDC_r_	DDC_r_
15	0.75	0.81	0.56
20	0.77	0.77	0.54
25	0.72	0.72	0.63
30	0.67	0.78	0.56
35	0.74	0.74	0.87
Mean	0.73	0.77	0.63

SDC_a_ = complementarity statistically downscaled to 10‐km scale from 50‐km atlas data. SDC_r_ = complementarity statistically downscaled to 10‐km scale from global range maps. DDC_r_ = complementarity directly downscaled to 10‐km scale from global range maps.

**Table 2:** Pearson's correlation coefficients of downscaled complementarity values, SDC_a_ (complementarity statistically downscaled to 10‐km scale from 50‐km atlas data), SDC_r_ (complementarity statistically downscaled to 10‐km scale from global range maps), and DDC_r_ (complementarity directly downscaled to 10‐km scale from global range maps), and “true” complementarity values of 5303 fine‐scale (10 × 10 km) cells in mainland Spain

	True	SDC_a_	SDC_r_
SDC_a_	**0.20**		
SDC_r_	**0.24**	**0.57**	
DDC_r_	**0.32**	**0.46**	**0.26**

Significant values are represented in bold. All correlations are significant at *P* < 0.05, using a modified *t*‐test that corrected for spatial autocorrelation (Dutilleul 1993).

## Discussion

For birds of Spain, statistically downscaled complementarity was about 75% as efficient as having full knowledge of species presence in its ability to improve on random selection of sites. Thus, statistically downscaled complementarity was a good surrogate for true complementarity, regardless of whether the coarse data were from 50 × 50 km atlas data or from global range maps. This suggests that the drivers of complementarity value at coarse (50‐km grid cell) scale – variables related to precipitation, NDVI, insolation, temperature, and seasonal variation in these variables (Table S1) – also drive complementarity value at fine (10‐km grid cell) scale. To our knowledge, this is the first study to model complementarity as a function of environmental variables at coarse resolution and downscale that model to predict complementarity at finer resolution. Thus, downscaled complementarity is fundamentally a new type of surrogate for species representation.

This finding is consistent with the recent demonstration that complementarity can be modeled as a function of a site's environmental variables for 11 datasets spanning global to local extents (Albuquerque and Beier 2015b,c; and in review). This finding is also consistent with a previous study that modeled species turnover and applied the model at a different resolution. In that study, Steinitz et al. (2005) modeled species similarity between pairs of 10 × 10‐m plots as a function of environmental variables and applied the resulting function to predict species similarity between 1 × 1 km cells; the observed similarity was highly correlated (*r* ^2^ = 0.67) with predicted similarity. The ability to upscale species turnover from 0.01 to 1 km^2^ (Steinitz et al. 2005) and to downscale complementarity from 50 × 50 km^2^ to 10 km^2^ (this study) suggests that complementarity may be generally predictable across grain sizes. However, we caution against making broad generalizations from two studies. Future studies using additional taxa across diverse settings, extents, and grain sizes are needed to determine the range of conditions over which complementarity can be rescaled.

Directly downscaled complementarity – interpreting range maps at the scale of a 10‐km grid – also performed well, with an efficiency of about 63%. This good performance was somewhat surprising because species do not occur at all locations within their mapped range (Hurlbert and White 2005), such that interpreting global range at resolutions smaller than 1° to 2° overestimates species richness by about 50% to 200% and distorts true patterns of species richness (Hurlbert and Jetz 2007). Nonetheless, the relative spatial patterns of RWR derived from this downscaling procedure reflected relative complementarity value of each cell reasonably well for birds of Spain.

The downscaled complementarity estimates produced by the three methods were only moderately correlated with each other (Table 2). This suggests that each procedure achieved similar levels of species representation by prioritizing sets of cells that only partially overlapped each other.

Conservation prioritization has been a challenge in areas with coarse or incomplete data on species distributions across sites (Ladle and Whittaker 2011). Our analyses suggest that downscaled complementarity might be an effective tool to prioritize sites for species representation in areas lacking high‐resolution biological data. DRWR joins two other new surrogates (predicted importance and predicted RWR) and one newly reinvigorated surrogate (environmental diversity) that can identify sites for species representation when a planner lacks data on species present in each site in the planning area (Table 3). Predicted importance (synonymous with predicted complementary) starts with species inventory data for a subset of sites in the planning area, uses Zonation to calculate complementarity, builds random forest models of the complementary value of each site as a function of freely available environmental variables, uses the model to predict complementarity for all sites, and uses these predicted values as a surrogate to prioritize all sites (Albuquerque and Beier 2015b). Predicted RWR (Albuquerque & Beier in review) is identical to predicted importance except that complementarity of the inventoried subset of sites is estimated by RWR instead of Zonation. Thus, the procedures for predicted importance and PRWR are the same as those for downscaled complementarity, except that the predicted importance and PRWR models extrapolate across sites instead of across scales. Environmental diversity (Faith and Walker 1996) requires no biotic data; instead, it quantifies abiotic environmental gradients as an ordination and selects sites that best span the ordination space.

**Table 3:** Alternative strategies to prioritize sites for species representation. “Direct selection” strategies require species inventories for 100% of sites in the planning area. The other strategies are surrogates, for use when a planner does not have inventories of species in all sites

Broad strategy	Specific strategy (key citations)	Biotic data required to use the surrogate (relative cost)	SAI for birds of Spain	Median SAI (# of study systems^a^)	Strengths and limitations
Direct selection for complementarity	Integer programming (Haight and Snyder 2009)	Inventory of every site (highest cost)	1.00	1.00	Proven to identify optimum solution.
	Marxan (Ardron et al. 2010)		~1.00^b^	~1.00^b^	Can integrate representation goal with other conservation goals (e.g., compactness, connectivity).
	Zonation (Moilanen et al. 2014)		~1.00^b^	~1.00^b^	
	RWR (Csuti et al. 1997; Albuquerque and Beier 2015a)		~1.00^c^	~1.00^c^ (12)	
Surrogates for complementarity	Downscaled complementarity (this article)	Coarse‐scale atlas or range maps (No cost for birds, mammals, amphibians. Availability of atlas data or range maps for other taxa varies among regions.)	0.63 to 0.75	N/A (1)	Not yet tested on other taxa or regions, or at scales <10 × 10 km cell.
	Predicted Importance, PI (Albuquerque and Beier 2015b)	Inventories for about 25% of sites (about 25% cost of direct selection)	0.69	0.50 (8)	CI for PI was about half as wide as for PRWR in tests on the same datasets.
	Predicted rarity‐weighted richness, PRWR (Albuquerque & Beier in review)	Inventories for about 20% of sites (about 20% cost of direct selection)	0.79	0.50 (6)	PRWR is easier and faster to compute than PI because no reserve selection software is needed.
	Environmental Diversity, ED (Faith and Walker 1996; Beier & Albuquerque 2015)	No biotic information required (no cost to acquire data)	0.26	0.40 (8)	May be useful to prioritize sites in a changing climate.

a Each study system is one taxonomic group in a particular study area; the number of tests refers to the tests reported in the references in column 2.

b Expected value (Moilanen et al. 2009).

c In tests against Marxan and Zonation.

In the one study system evaluated by all four surrogates (birds of Spain), the SAI for downscaled complementarity was as high as that of predicted importance and PRWR, and data acquisition cost was zero (Table 3). Thus, with the recent release of global range maps for birds (BirdLife International 2012), and amphibians and mammals (IUCN Red List Spatial Data, IUCN, Gland, Switzerland; available at: http://www.iucnredlist.org/technical-documents/spatial-data), downscaled complementarity could be applied widely for these taxa. Our study justifies additional tests to determine whether downscaled complementarity is an effective surrogate for other regions and taxa. One difficulty in conducting such tests will be finding reasonably independent sets of biodiversity data, including fine‐scale species inventories. One limitation of downscaled complementarity is that comprehensive atlases for invertebrates and plants are not available for most regions. Although a surrogate for some vertebrate groups is better than no surrogate, a conservation prioritization that ignores plants and invertebrates is far from comprehensive.

Another crucial need is for tests at spatial resolution finer than 10 × 10 km cells. The performance of surrogates at finer scales will likely differ from what is reported in Table 3. Until such tests have been completed, we caution against assuming that any surrogate can reliably prioritize sites for species representation.

All four surrogates listed in Table 3 require additional development, including representation goals >1 occurrence per species, goals that vary among species, prioritizing sites to expand an existing reserve network (rather than prioritizing on a blank map), consideration of site‐specific costs of conservation, and integration of species representation goals with conservation goals for compactness, connectivity, and ecological and evolutionary processes (Margules and Pressey 2000).

Much work remains to be made to evaluate approaches to prioritize sites for species representation in a planning area when a planner has limited information on species present in those sites, and to integrate species representation with other conservation goals. We are pleased that over the last couple years, there has been a proliferation of promising approaches, and we hope that one or more of these approaches will soon prove broadly useful in conservation‐relevant contexts.

## Conflict of Interest

None declared.

## Supporting information


**Table S1.** Loadings (correlations) of environmental variables of the 6 first factors of the varimax‐rotated factor analysis, and percent of variance explained by each factor for 241 coarse (50 × 50 km) cells in Spain.
