# Supplementary material for: Downscaling patterns of complementarity to a finer resolution and its implications for conservation prioritization
Source: Ecol Evol. 2016 May 18;6(12):4032–40. doi: 10.1002/ece3.2190 (PMC4972229; doi:10.1002/ece3.2190)
Supplement: Supplementary file 1 — Table S1. Loadings (correlations) of environmental variables of the 6 first factors of the varimax‐rotated factor analysis, and percent of variance explained by each factor for 241 coarse (50 × 50 km) cells in Spain. [file ECE3-6-4032-s001.docx]

**Supporting Information**

Table S1. Loadings (correlations) of environmental variables of the 6 first factors of the varimax-rotated factor analysis, and percent of variance explained by each factor for 241 coarse (50x50 km) cells in Spain. Bold font indicates loadings > 0.60 or < -0.60. Variables used to downscale rarity-weighted richness are marked as *.

| **Environmental variables** | Principal Component Factors | | | | | |
| --- | --- | --- | --- | --- | --- | --- |
|  | I | II | III | IV | V | VI |
| **Energy related variables** |  |  |  |  |  |  |
| Annual mean temperature | **0.871** | -0.296 | -0.026 | -0.251 | -0.201 | 0.030 |
| Mean diurnal temperature range | 0.320 | 0.329 | **0.723** | -0.072 | 0.432 | 0.116 |
| Isothermality* | 0.208 | -0.024 | -0.290 | -0.171 | **0.657** | -0.290 |
| Temperature seasonality | 0.160 | 0.329 | **0.841** | 0.009 | 0.053 | 0.234 |
| Max temperature of warmest month | **0.779** | 0.057 | 0.495 | -0.193 | 0.066 | 0.197 |
| Min temperature of coldest month | 0.686 | -0.399 | -0.471 | -0.224 | -0.245 | -0.020 |
| Annual temperature range | 0.258 | 0.339 | **0.809** | -0.024 | 0.237 | 0.203 |
| Mean temperature of wettest quarter | 0.185 | -0.417 | 0.314 | -0.203 | **-0.605** | -0.263 |
| Mean temperature of driest quarter | **0.730** | -0.146 | 0.058 | -0.183 | 0.376 | 0.108 |
| Mean temperature of warmest quarter | **0.862** | -0.148 | 0.300 | -0.213 | -0.152 | 0.115 |
| Mean temperature of coldest quarter | **0.772** | -0.400 | -0.317 | -0.220 | -0.203 | -0.057 |
| Hours of sunshine average | 0.487 | -0.302 | 0.248 | **-0.735** | -0.018 | -0.101 |
| Hours of sunshine maximum* | -0.292 | -0.116 | 0.129 | **-0.917** | 0.066 | 0.076 |
| Hours of sunshine minimum | **0.736** | -0.255 | 0.194 | -0.111 | -0.121 | -0.256 |
| Hours of sunshine first quartile | **0.875** | -0.199 | 0.268 | -0.027 | 0.023 | 0.008 |
| Hours of sunshine fourth quartile | 0.308 | -0.163 | 0.148 | **-0.882** | -0.037 | -0.093 |
| Hours of sunshine interquartile | **-0.845** | 0.165 | -0.243 | -0.230 | -0.036 | -0.036 |
| Hours of sunshine range | **-0.716** | 0.120 | -0.066 | -0.462 | 0.128 | 0.234 |
| **Water** | | | | | | |
| Annual mean precipitation | -0.362 | 0.043 | **-0.889** | 0.141 | 0.076 | 0.059 |
| Precipitation of wettest month | -0.145 | -0.019 | **-0.948** | 0.111 | 0.079 | -0.014 |
| Precipitation of driest month | **-0.856** | 0.001 | -0.386 | 0.155 | -0.122 | 0.005 |
| Precipitation seasonality* | **0.927** | -0.063 | -0.177 | 0.080 | 0.110 | 0.100 |
| Precipitation of wettest quarter* | -0.094 | 0.008 | **-0.954** | 0.115 | 0.123 | 0.018 |
| Precipitation of driest quarter | **-0.847** | -0.023 | -0.424 | 0.109 | -0.120 | 0.007 |
| Precipitation of warmest quarter | **-0.831** | -0.083 | -0.426 | 0.097 | -0.213 | -0.030 |
| Precipitation of coldest quarter | 0.041 | 0.082 | **-0.929** | 0.102 | 0.235 | 0.072 |
| **Vegetation** | | | | | | |
| NDVI (normalized difference vegetation index) average | -0.143 | **0.975** | 0.030 | 0.073 | 0.036 | 0.110 |
| NDVI maximum | -0.113 | **0.964** | 0.053 | 0.069 | 0.031 | 0.206 |
| NDVI minimum* | -0.156 | **0.977** | 0.030 | 0.056 | 0.036 | -0.003 |
| NDVI of the first quartile | -0.172 | **0.973** | 0.018 | 0.075 | 0.039 | 0.039 |
| NDVI of the fourth quartile | -0.117 | **0.966** | 0.042 | 0.073 | 0.033 | 0.193 |
| NDVI interquartile* | 0.189 | 0.448 | 0.130 | 0.025 | -0.010 | **0.799** |
| NDVI range | 0.068 | 0.529 | 0.100 | 0.081 | 0.002 | **0.769** |
| **Topography** |  |  |  |  |  |  |
| Range in elevation | -0.544 | 0.438 | 0.394 | 0.427 | 0.294 | 0.056 |
| Mean slope | -0.459 | 0.320 | -0.193 | **0.754** | 0.030 | 0.095 |
| Aspect diversity | -0.226 | **0.872** | 0.064 | 0.367 | 0.059 | 0.137 |
| Topographic diversity | 0.011 | **0.882** | 0.256 | 0.095 | -0.004 | -0.001 |
